# A retrospective survival analysis of Glioblastoma patients treated with selective serotonin reuptake inhibitors

**DOI:** 10.1016/j.bbih.2019.100025

**Published:** 2019-12-16

**Authors:** Sebastian Otto-Meyer, Rian DeFaccio, Corey Dussold, Erik Ladomersky, Lijie Zhai, Kristen L. Lauing, Lakshmi R. Bollu, Christina Amidei, Rimas V. Lukas, Denise M. Scholtens, Derek A. Wainwright

**Affiliations:** a Department of Neurological Surgery, Chicago, IL, 60611, USA; b Department of Preventative Medicine-Biostatistics, Chicago, IL, 60611, USA; c Department of Neurology, Chicago, IL, 60611, USA; d Robert H. Lurie Comprehensive Cancer Center of Northwestern University, Chicago, IL, 60611, USA; e Department of Medicine-Division of Hematology and Oncology, Chicago, IL, 60611, USA; f Department of Microbiology-Immunology, Northwestern University Feinberg School of Medicine, Chicago, IL, 60611, USA

**Keywords:** Psychosocial, Biobehavioral, Glioma, Depression, Antidepressant, Immunosuppression

## Abstract

Glioblastoma (GBM) is the most common and aggressive form of malignant glioma in adults with a median overall survival (OS) time of 16–18 months and a median age of diagnosis at 64 years old. Recent work has suggested that depression and psychosocial distress are associated with worse outcomes in patients with GBM. We therefore hypothesized that the targeted neutralization of psychosocial distress with selective serotonin reuptake inhibitor (SSRI) antidepressant treatment would be associated with a longer OS among patients with GBM. To address this hypothesis, we retrospectively studied the association between adjuvant SSRI usage and OS in GBM patients treated by Northwestern Medicine-affiliated providers. The medical records of 497 GBM patients were analyzed after extraction from the Northwestern Medicine Enterprise Data Warehouse. Data were retrospectively studied using a multivariable Cox model with SSRI use defined as a time-dependent variable for estimating the association with OS. Of the 497 patients, 315 individuals died, while 182 were censored due to the loss of follow-up or were alive at the end of our study. Of the 497 patients, 151 had a recorded use of SSRI treatment during the disease course. Unexpectedly, SSRI usage was not associated with an OS effect in both naïve (HR = 0.81, 95% CI = 0.64–1.03) and adjusted time-dependent (HR = 1.26, 95% CI = 0.97–1.63) Cox models. Ultimately, we failed to find an association between SSRI treatment and an improved OS of patients with GBM. Additional work is necessary for understanding the potential therapeutic effects of SSRIs when combined with other treatment approaches, and immunotherapies in particular, for subjects with GBM.

## 1. Introduction

Glioblastoma (GBM) is the most common form of malignant glioma, with a median overall survival (OS) time of 16–18 months in adults (Koshy et al., 2012; Stupp et al., 2017). Patients with GBM are poorly responsive to conventional therapy, which includes maximal surgical resection when possible combined with radiation treatment, chemotherapy with the DNA-alkylating drug temozolomide, and more recently, tumor treating fields. Due to the persistently grim prognosis associated with GBM, experimental approaches evaluating diverse immunotherapeutic treatments including vaccines, immune checkpoint inhibitors, and CAR T-cells are actively under clinical study. However, to-date, these methods have failed to demonstrate improvement in GBM patient OS among phase III clinical trials (Weller et al., 2017; Reardon et al., 2017; Schuster et al., 2015; Bloch et al., 2017), motivating research into adjunct treatments that may improve survival through a synergistic mechanism (Ladomersky et al., 2018; Tan et al., 2018).

Reversing depressive symptoms and/or psychological distress may improve the OS of patients with GBM (Otto-Meyer et al., 2019). Depression and psychological distress are relatively common among individuals with cancer, although the degree and severity vary with the malignant cell of origin (Krebber et al., 2014). Strikingly, depression is more common among individuals with primary brain tumors as compared to other cancer diagnoses, with a recent study estimating that depression affects nearly 36 percent of brain cancer patients (Hartung et al., 2017). Importantly, however, this number fails to capture patients experiencing psychological distress that does not meet the full criteria for a diagnosis of depression – potentially contributing to an underestimation of the full impact of maladaptive psychological effects during cancer treatment (Liu et al., 2018). Indeed, mental health needs are often unmet in patients with primary brain tumors (Langbecker and Yates, 2016). Increased psychological distress not only reduces patient quality of life, but also potentially affects survival outcomes. A recent meta-analysis found a decrease in OS among patients with high-grade glioma who were diagnosed with depression (Shi et al., 2018). This observation was supplemented by an independent analysis revealing decreased OS of patients with high-grade glioma that were diagnosed with preoperative depression (Gathinji et al.).

If depressive-like symptoms contribute to a worse overall prognosis in patients with GBM, then OS might theoretically be improved with a therapeutic approach aimed at reversing the maladaptive causes. A common method for treating depression and other forms of psychological distress is through the use of selective serotonin reuptake inhibitor (SSRI) antidepressants. Despite limited data in patients with cancer, SSRIs are often used to treat depression in these individuals due to their favorable safety profile and previous study results supporting their general use (Ostuzzi et al., 2018). This class of medication may help to address the negative impact on OS as mediated by the pervasive psychological distress that is characteristic of patients with GBM. Additionally, some studies suggest a potential alternative mechanism of action for SSRIs, which is mediated through a direct cytotoxic effect on the tumor cells to improve the survival of GBM patients (Liu et al., 2015; Chen et al., 2018; Then et al., 2017). Finally, an improved mood due to the effects of SSRI treatment may improve immunological control of GBM by decreasing the risk of tumor progression that results from the psychological distress that is mediated by immune dysregulation (Powell et al., 2013; Qiao et al., 2018).

The impact of SSRIs on GBM patient OS was previously studied during a retrospective analysis of 160 individuals at the Mayo Clinic (Caudill et al., 2011) suggesting that a potential survival benefit existed in those individuals prescribed antidepressants. To extend and validate this investigation further, we retrospectively studied a 497 patient cohort diagnosed and treated for primary GBM at Northwestern Medicine. Using a multivariable Cox model with time-dependent covariate specification, we examined the association between SSRI use and OS. We hypothesized that SSRI use would be associated with improved OS in GBM patients as compared to untreated peers. In contrast to the work from previous groups and to our own hypothesis, we did not find an association between SSRI use and an improved GBM patient OS as compared to untreated individuals.

## 2. Materials and methods

### 2.1. Inclusion criteria

**Fig. 1.**
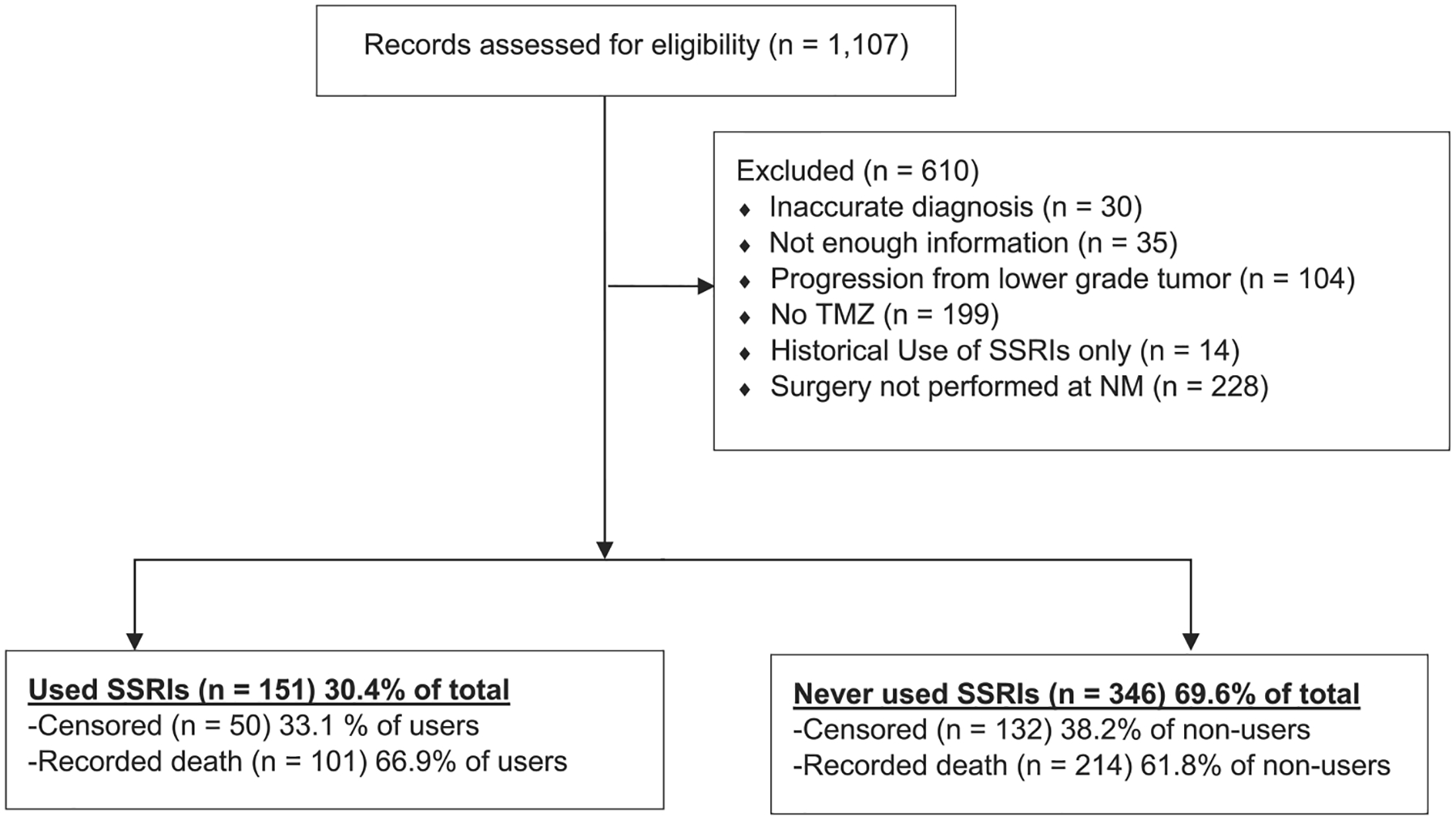
Inclusion and exclusion criteria for the analysis of subjects diagnosed with glioblastoma. Patient exclusions are noted and the division of patients who used SSRIs versus those who did not are summarized. Censored patients had no recorded death date and were censored at the date of their last appointment or, if they were still alive, at study end. TMZ, temozolomide; SSRI, selective serotonin reuptake inhibitor; NM, Northwestern Medicine.

The data set was comprised of patient records from the Northwestern Medicine Enterprise Data Warehouse (EDW) with a diagnosis of GBM between January 1, 2000 until March 8, 2018. A diagnosis was based on the neuropathological confirmation of GBM from surgically-resected specimens. As shown in Fig. 1, the initial dataset included 1,107 patient records with a diagnosis of GBM listed. The records were manually reviewed for accuracy and the exclusion process is included in Fig. 1. Patients were excluded based on the following criteria: (i) an inaccurate diagnosis recorded in the EDW (n = 30); (ii) not enough information included in the patient record for positively confirming a diagnosis or patient course of treatment (n = 35); (iii) a concurrent diagnosis of neurofibromatosis-1 (n = 2); (iv) a previously confirmed diagnosis of a lower grade tumor, with or without confirmation of subsequent progression to GBM (n = 102); (v) a lack of physician notes positively indicating evidence of treatment with temozolomide (TMZ) (n = 199); (vi) a historical usage of SSRIs prior to a GBM diagnosis, with no evidence of treatment post-diagnosis (n = 14); and (vii) a diagnosis at clinical centers other than Northwestern Medicine, as this may have led to missing patient record information, including all possible dates of SSRI use (n = 228). Exclusions were performed in a sequential prioritization order as listed above. This study was granted exemption status from the Northwestern University Institutional Review Board based on the de-identified GBM patient records provided for analysis.

### 2.2. NM EDW-Obtained variables

The final cohort obtained for analysis included 497 GBM patients between the ages of 18 and 91 years of age. Demographic characteristics and clinical information collected included: (i) the age at time of diagnosis; (ii) sex; (iii) race; and (iv) type of surgical intervention (biopsy versus resection). The Charlson Comorbidity Index (CCI) score was calculated as a weighted sum of 16 common comorbidities for predicting the risk of death in hospitalized patients (Austin et al., 2015). The CCI score was calculated without age adjustment, since age was already controlled for during the analyses. Intermediate follow-up data included the start and stop times of SSRI usage. Survival was determined from the date of initial diagnosis based on pathological characterization, with the endpoint defined as the date of all-cause death. Observations for which the patient was alive at last point of follow-up were censored during the statistical analysis. SSRI use was searched for within the medication records of the EDW, and included both inpatient administration and reported outpatient prescriptions. SSRI search terms included the generic and primary brand name used in the United States and are as follows: (i) fluoxetine, Prozac; (ii) citalopram, Celexa; (iii) escitalopram, Lexapro; (iv) sertraline, Zoloft; (v) paroxetine, Paxil; (vi) vilazodone, Viibyrd. Start and stop times for SSRI use were determined by relying on medication records within the EDW. If multiple SSRIs were sequentially used over the duration of treatment, start time was defined as the first use of any SSRI, while the stop time was defined as the last use of any SSRI.

### 2.3. Statistical analysis

An event chart was constructed to visualize the relationship between SSRI treatment and OS time using the ‘Hmisc’ R package (Suissa, 2008). Univariable associations between demographic characteristics and OS in GBM patients were examined using Cox Proportional Hazards (Cox PH) models. The unadjusted association of SSRI treatment with OS was examined in a univariable Cox model with SSRI use modeled as a time-dependent covariate. Time-dependent modeling of SSRI use was critical to avoid the “immortal time bias”, given that the SSRI use status varied over the follow-up interval and was not fixed from the time of initial diagnosis. The effect of immortal time bias is often significant in magnitude and it is for this reason that three commonly used types of models were evaluated to determine the robustness of findings (Cho et al., 2017; Agarwal et al., 2018). Adjusted Cox models included predictors that were significantly associated with OS in a univariable analysis. For these and all other statistical tests, significance was defined by *p* < 0.05. These covariates, in addition to sex, were included in the fully-adjusted Cox model. Sex was included in the multivariable models because of a clinical acceptance that it is related to OS time (Tian et al., 2018). All Cox models and related analyses were performed using the ‘survival’ R package (Terry M Therneau, 2018).

A doubly robust Cox model was fit using inverse probability of treatment weights (IPW weights) and inverse probability of censoring weights (IPC weights) with the ‘IPW’ R package (Willem and van der Wal, 2011). The weights were calculated separately and multiplied together in the manner described by Geskus and van der Wal (Willem and van der Wal, 2011). In addition, landmark analyses were performed as a third approach to confirm the robustness of findings. For descriptive purposes, the hazard ratios from the 1st quartile, median, and 3rd quartile of follow-up are presented in Table 1. All statistical analysis was completed using R version 3.5.1 (Team, 2013).

## 3. Results

### 3.1. Baseline characteristics

**Fig. 2.**
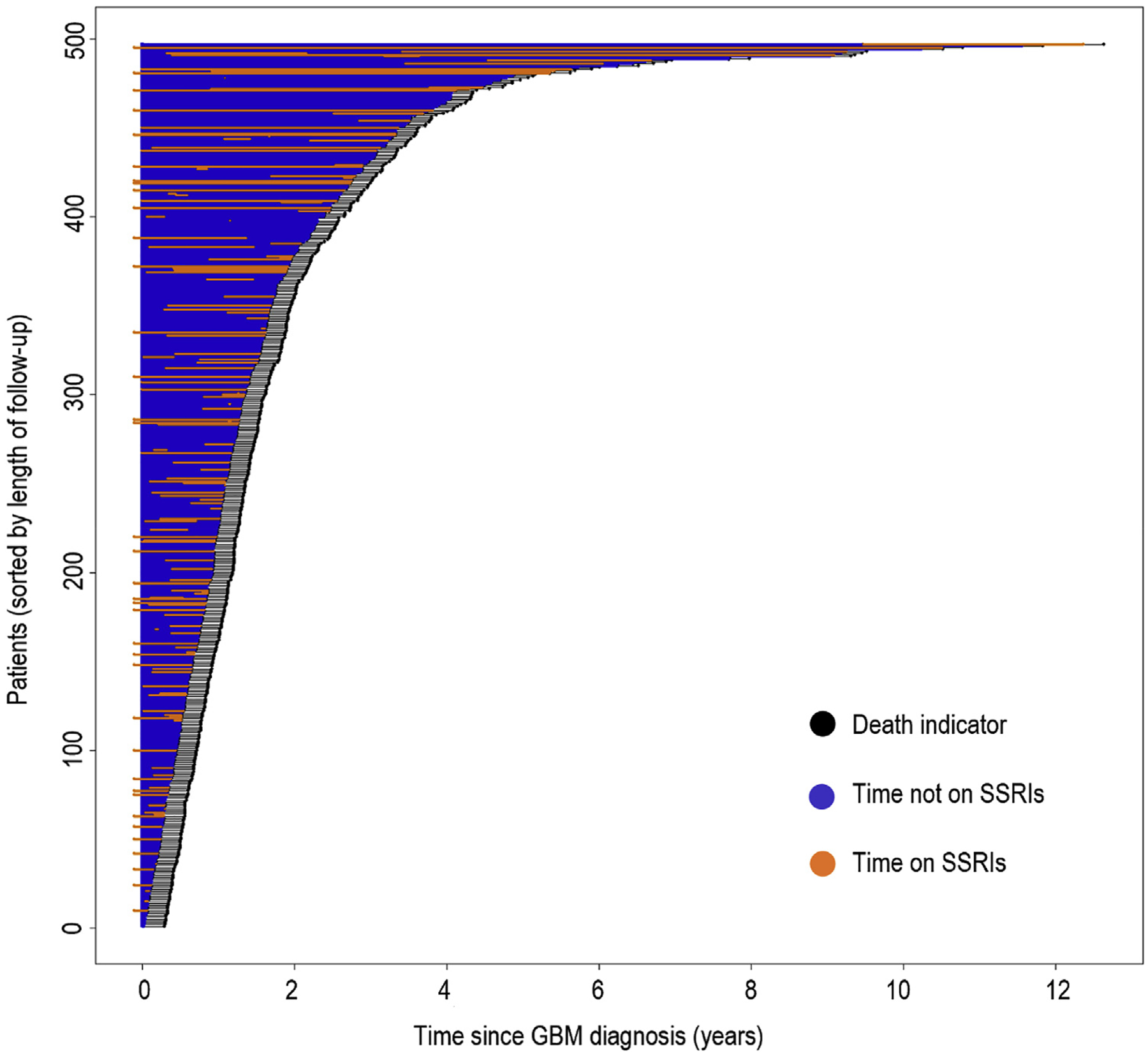
Distribution of SSRI use in patients. Patients were sorted by length of time to censor or death. For each patient: death is indicated by a black dot; time on SSRI is indicated by an orange line; time not on SSRI is indicated by a blue line. No clear pattern is visible between length of time on SSRI and length of follow-up.

The relationship between SSRI ever-use after diagnosis and baseline patient traits are reported in Table 2. There were 497 patients in total, with 151 prescribed an SSRI post-GBM diagnosis and 346 with no record of SSRI use post-diagnosis. GBM patients who used SSRIs were more likely to have also experienced a tumor resection rather than a biopsy, as compared to SSRI non-users. SSRI users also had differences in sex and follow-up status (censoring vs. recorded death). Race and CCI score were not significantly different among SSRI users and non-users. Among the SSRI users, the median time until SSRI prescription after GBM diagnosis was 85 days. The median time spent on SSRIs among users was 296 days. The median follow-up time among all patients was 395 days. Fig. 2 displays an event chart that failed to show an obvious visual association or trend between follow-up and SSRI use.

### 3.2. Unadjusted analysis

A naïve analysis was performed first, with SSRI use treated as a baseline covariate (an indicator of SSRI ever-use). Although the hazard ratio from this analysis suggested a positive association, it was not significant (HR: 0.81, 95% CI: 0.64–1.03). Further analyses incorporated SSRI use as a time-dependent covariate as described in the Materials and Methods section (see Table 1 for hazard ratios). Type of surgical intervention, age at the time of diagnosis, and SSRI use were found to have significant associations with OS among univariable analyses. In the unadjusted model, SSRI use as a time-dependent variable was associated with a 34% higher hazard of death as compared to SSRI non-use (HR: 1.34, 95% CI: 1.04–1.72). Patients receiving resection as opposed to biopsy had a 50% lower hazard of death (HR: 0.5, 95% CI: 0.38–0.66). Since type of surgical operation was associated with both SSRI treatment and survival, it was considered as a potential confounder. However, it was not significant during multivariable analysis and did not affect the hazard ratio estimate for the association between SSRI use and OS. A violation of the proportional hazards assumption while utilizing the univariable Cox model of hazard by age at the time of diagnosis was remedied by the introduction of a time-dependent coefficient for the association between subject age and OS. The resulting model had a slightly higher hazard ratio for the association between age and OS during the first 253 days of follow-up and a lower hazard ratio after 253 days.

### 3.3. Adjusted analysis

**Fig. 3.**
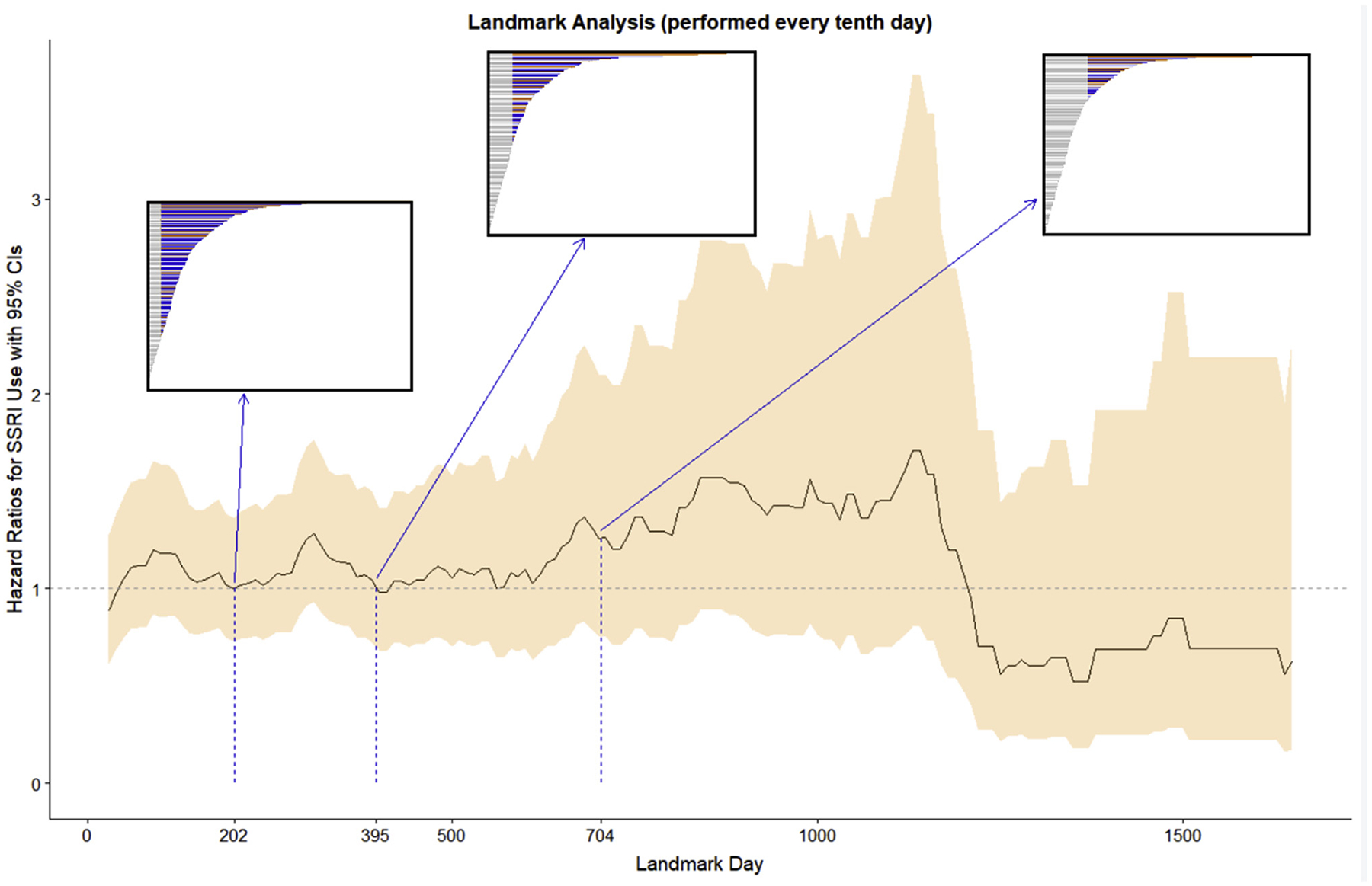
Trends in landmark analysis hazard ratios. The analyses presented in Table 1 were performed at the 1st quartile (202 days), median (395 days), and 3rd quartile (704 days) of follow-up. The boxes show follow-up times for all patients with the subset of the sample used in each of the final analyses colored red and blue, indicating SSRI use up to the landmark time.

The two significant covariates from the univariate analyses included surgical intervention and patient age and were chosen, in addition to sex, for the adjustment of the multivariable analysis. The hazard ratio for mortality risk associated with SSRI use in the time-dependent multivariable Cox model was 1.26 (95% CI: 0.97–1.63), as compared to SSRI non-use. The three landmark analyses presented in Table 1 and Fig. 3 were performed at the landmark times of 202, 395, and 704 days post-diagnosis, which correspond with the 1st quartile, median, and 3rd quartile of follow-up. The hazard ratios for the three landmark analyses were 1.01 (95% CI: 0.74–1.38), 1.05 (95% CI: 0.73–1.5), and 1.26 (95% CI: 0.75–2.09). The hazard ratio from the weighted Cox model was 1.06 (95% CI: 0.8–1.4).

## 4. Discussion

Using a time-dependent Cox model, we discovered that patients with GBM treated with SSRIs did not have an associated improvement of OS as compared to untreated GBM patients. This finding differs from our original hypothesis, as well as the implications of the previous study by Caudill et al. (2011), which indicated a potential increase in OS among GBM patients that had used SSRIs. There are several factors that may explain the conflicting conclusions between our study and the study by Caudill et al. First, our investigation is significantly larger with respect to the number of patients analyzed, which increases its likelihood to estimate a true effect. Additionally, we used time-dependent modeling that allows for the estimate of impact by SSRI treatment, while avoiding immortal time bias – a methodology that is well-established in the literature (Busby et al., 2018). Finally, our inclusion criteria were different than the study by Caudill et al., as we only analyzed patients who had been co-treated with temozolomide.

Although our results indicate no association between SSRIs and OS, there are several considerations merited by these findings. First, patients treated with SSRIs may have had a worse overall prognosis. Increased psychosocial distress is associated with worse overall health and quality of life in neurosurgical and glioma patients (Hoffmann et al., 2017; Hickmann et al., 2017), implying that the individuals treated with SSRIs for addressing maladaptive levels of distress may have also had worse health and functioning that was not captured during our covariate analyses. If this was the case, it may have also countered any potential benefit from SSRIs during our investigation. Additionally, the lack of a negative association with OS in adjusted models provides an indication that SSRIs do not cause harm to patients with GBM. This is a particularly significant and potentially important point, since the high prevalence of depression and anxiety (Shi et al., 2018; Huang et al., 2017), as well as the current difficulty in providing strong recommendations for the treatment of GBM patients, is partly due to the lack of randomized trials and data in the field (Ostuzzi et al., 2018; Pace et al., 2017). Although SSRIs are generally benign drugs, GBM provides a unique setting for their use based on the neuroanatomical tumor location inside of the brain and/or spinal cord. Neurological insult and inflammation is persistently present in GBM and has been linked to the pathogenesis of depression, potentially contributing to a complex interplay of etiologies that exacerbate and/or mediate psychological distress in GBM patients (Miller, 2016; Yeung et al., 2013; Chiorean et al., 2014). The ultimate effects of SSRIs may therefore be altered in GBM patients as compared to individuals diagnosed with cancer arising and/or residing outside of the central nervous system. It is therefore important to highlight that the present study failed to find a negative association between SSRI use and OS.

Our investigation relied upon GBM patients who were diagnosed and received at least part of their treatment within a single health care system. While this may have limited the generalizability of our study, it also enabled us to ensure the reliability of diagnosis and access to detailed medical records, which avoided a potential recall bias and limited confounders from different medical treatment centers. Furthermore, we were able to limit the dataset to patients who were treated with temozolomide – the current standard of care chemotherapeutic drug prescribed to all GBM patients – which allowed for standardization of the study population (Stupp et al., 2005).

Aside from the causality concerns inherent among retrospective analyses, there were several limitations associated with our investigation. First, initial performance status could not be assessed in this population, which is traditionally performed in GBM patients by assessing the Karnofsky Performance Status (KPS) or ECOG Performance Status scores (Kelly and Shahrokni, 2016). These scores were recorded sporadically among physicians’ notes and at various durations of time from the initial diagnosis. Second, if GBM patients received a prescription from a provider outside of Northwestern Medicine, this may have been inconsistently recorded. However, by limiting our dataset to patients who received their diagnosis at Northwestern Medicine and with whom we have evidence of standard of care TMZ treatment, there is a reasonable level of confidence that most prescriptions were captured within our study. Finally, and partially due to the prolonged time span of our retrospective dataset, we were unable to reliably capture prognostic molecular markers including wild-type versus mutant isocitrate dehydrogenase status, as well as promoter methylation status of O (Bloch et al., 2017)-methylguanine-DNA methyltransferase that have emerged as major prognostic factors of GBM patient OS (Hegi et al., 2005; SongTao et al., 2012).

Although SSRI use was not associated with an improved OS in our study, the use of SSRIs in GBM patients and animal models merits further investigation. Psychosocial and biobehavioral stress, and their associated downstream signaling processes, have been associated with a decreased anti-tumor immune response and increased tumor growth in mouse models of cancer arising or residing outside of the brain (Qiao et al., 2018; Schmidt et al., 2016; Nissen et al., 2018). While blocking beta-adrenergic signaling has been a major target of this research thus far, decreasing the levels of psychosocial distress, as well as reducing stress signaling pathways through SSRI treatment, may be viable future treatment options (Wang et al., 2018; Montoya et al., 2017; Kokolus et al., 2018). If immunological function can be rescued with SSRI treatment, it may serve as an adjunct approach for improving patient responsiveness to immunotherapy – the latter of which has yet to show any GBM patient survival benefit among phase III clinical trials to-date. Understanding how SSRIs affect the anti-brain tumor immune response, as well as the host immune response to immunotherapy while evaluating preclinical models, may also provide rationale for future prospective clinical evaluation and should be considered a priority. Ultimately, although the ability to statistically analyze the interaction between SSRI use and immunotherapy treatment in GBM patients is not possible in our current dataset, it is an exciting prospect and included in our future directions through cooperative group efforts.

## Figures and Tables

**Table 1:** Hazard ratios (HR) for death among GBM patients.

	HR (95% CI)
Age at Diagnosis	
Follow-up < 253 days	1.05 (1.03–1.07)
Follow-up > 253 days	1.02 (1.01–1.03)
Sex	
Male	1.0 (reference)
Female	0.95 (0.76–1.19)
Operation	
Biopsy	1.0 (reference)
Resection	0.5 (0.38–0.66)
CCI Score	
0	1.0 (reference)
1	0.87 (0.63–1.22)
2	1.37 (0.85–2.22)
3+	1.05 (0.57–1.92)
Race	
White	1.0 (reference)
Asian	0.51 (0.19–1.37)
Black	1.02 (0.63–1.64)
Other	1.0 (0.68–1.45)
Declined	1.01 (0.68–1.50)
SSRI (naive analysis)^a^	
No	1.0 (reference)
Yes	0.81 (0.64–1.03)
SSRI (unadjusted, time-dependent)	
No	1.0 (reference)
Yes	1.34 (1.04–1.72)
SSRI (adjusted, time-dependent)^b^	
No	1.0 (reference)
Yes	1.27 (0.98–1.64)
SSRI (Landmark Analysis at 202 days)^c^	
No	1.0 (reference)
Yes	1.01 (0.74–1.38)
SSRI (Landmark Analysis at 395 days)^c^	
No	1.0 (reference)
Yes	1.05 (0.73–1.50)
SSRI (Landmark Analysis at 704 days)^c^	
No	1.0 (reference)
Yes	1.26 (0.75–2.09)
SSRI (Weighted Cox Model)^d^	
No	1.0 (reference)
Yes	1.06 (0.8–1.4)

a Treating SSRI ever-use as a baseline variable.

b Adjusted for sex, operation, and age at diagnosis.

c Adjusted for sex, operation, and age at diagnosis. SSRI status landmarked.

d Using IPC and IPT weights. Adjusted for residual confounding by operation and age at diagnosis.

**Table 2:** Characteristics of study patients according to SSRI use.

		SSRI Use	
	Total (n = 497)	No: n = 346 (69.6%)	Yes: n = 151 (30.4%)
Age (years)^a^			
Mean (SD)	59.3 (14.0)	59.1 (14.0)	59.7 (13.9)
Sex			
Male	299 (60.2%)	216 (62.4%)	83 (55.0%)
Female	198 (39.8%)	130 (37.6%)	68 (45.0%)
Race			
White	366 (73.6%)	249 (72.0%)	117 (77.5%)
Asian	9 (1.8%)	6 (1.7%)	3 (2.0%)
Black	26 (5.2%)	20 (5.8%)	6 (4.0%)
Other	47 (9.5%)	37 (10.7%)	10 (6.6%)
Declined	49 (9.9%)	34 (9.8%)	15 (9.9%)
Operation			
Biopsy	86 (17.3%)	71 (20.5%)	15 (9.9%)
Resection	411 (82.7%)	275 (79.5%)	136 (90.1%)
CCI Score^b^			
0	384 (77.3%)	272 (78.6%)	112 (74.2%)
1	66 (13.3%)	40 (11.6%)	26 (17.2%)
2	28 (5.6%)	20 (5.8%)	8 (5.3%)
3+	19 (3.8%)	14 (4.0%)	5 (3.3%)

a Age at diagnosis of GBM.

b CCI = Charlson Comorbidity Index.
